# Serum proteomics unveil characteristic protein diagnostic biomarkers and signaling pathways in patients with esophageal squamous cell carcinoma

**DOI:** 10.1186/s12014-022-09357-x

**Published:** 2022-05-24

**Authors:** Wenhu Liu, Qiang Wang, Jinxia Chang, Anup Bhetuwal, Nisha Bhattarai, Fan Zhang, Jiancai Tang

**Affiliations:** 1 School of Pharmacy, School of Basic Medical Sciences & Forensic Medical, North Sichuan Medical College, Nanchong, China; 2 Department of Clinical Laboratory, Translational Medicine Research Center, Affiliated Hospital of North Sichuan Medical College, Nanchong, China; 3 Sichuan Key Laboratory of Medical Imaging, North Sichuan Medical College, Nanchong, China; 4 Department of Neurology, North Sichuan Medical College, Nanchong, China

**Keywords:** Esophageal squamous cell carcinoma, Serum proteomics, Biomarker panel, Signaling pathway

## Abstract

**Background:**

Esophageal squamous cell carcinoma (ESCC) is a common digestive tract malignant tumor with high incidence and dismal prognosis worldwide. However, the reliable biomarkers for clinical diagnosis and the underlying signaling pathways insights of ESCC are not unequivocally understood yet. The serum proteome may provide valuable clues for the early diagnosis of ESCC and the discovery of novel molecular insights.

**Methods:**

In the current study, an optimized proteomics approach was employed to discover novel serum-based biomarkers for ESCC, and unveil abnormal signal pathways. Gene ontology (GO) enrichment analysis was done by Gene Set Enrichment Analysis (GSEA) and Metascape database, respectively. Pathway analysis was accomplished by GeneCards database. The correlation coefficient was assessed using Pearson and distance correlation analyses. Prioritized candidates were further verified in two independent validation sets by enzyme-linked immunosorbent assay (ELISA) and immunohistochemistry (IHC) staining.

**Results:**

A total of 633 non-redundant proteins were identified in the serum of patients with ESCC, of which 59 and 10 proteins displayed a more than 1.5-fold increase or decrease compared with healthy controls. Verification was performed for six candidate biomarkers, including S100A8/A9, SAA1, ENO1, TPI1 and PGAM1. Receiver operating characteristics (ROC) curve plotting showed the high diagnostic sensitivity and specificity of these six protein molecules as a biomarker panel: the area under characteristic curve (AUC) is up to 0.945. Differentially expressed proteins were subjected to functional enrichment analysis, which revealed the dysregulation of signaling pathways mainly involved in glycolysis, TLR4, HIF-1*α*, Cori cycle, TCA cycle, folate metabolism, and platelet degranulation. The latter finding was all the more noteworthy as a strong positive correlation was discovered between activated glycolysis and TLR4 pathways and unfavorable clinicopathological TNM stages in ESCC.

**Conclusions:**

Our findings propose a potential serum biomarker panel for the early detection and diagnosis of ESCC, which could potentially broaden insights into the characteristics of ESCC from the proteomic perspective.

**Supplementary Information:**

The online version contains supplementary material available at 10.1186/s12014-022-09357-x.

## Introduction

Esophageal squamous cell carcinoma (ESCC), the major histopathological subtype of esophageal cancer, is one of the most lethal malignancies of digestive tumors worldwide, with a 5-year survival rate of less than 20% [1, 2]. The pathogenesis of ESCC is concealed due to the lack of specific signs or symptoms at the early stage, but once the patients start to display clinical symptoms, they would have already developed a locally advanced or metastatic tumor and missed the optimal treatment period. Therefore, the poor clinical outcome of ESCC is the most dismal among digestive tumors. Despite the advances in therapeutic strategies, early detection and diagnosis of ESCC still faces difficulty in clinical practice, mainly due to the lack of highly sensitive and specific diagnostic protein biosignature, and many unknowns of its biological characteristics.

Several potential tumor biomarkers have been reported in various biofluids of ESCC, such as tissue, blood and urine. Cui et al. demonstrated that Profilin 2 (PFN2) expression was markedly increased in tissues of ESCC patients compared with controls. Its role in promoting ESCC progression, metastasis and portending poor prognosis suggests the potential of PFN2 as an early biomarker [3]. Serum HOX transcript antisense RNA (HOTAIR) was reported to be correlated with pathological TNM stages, which might serve as a potential biosignature for the diagnosis of ESCC [4]. Five miRNAs (miR-1273f, miR-619-5p, miR-150-3p, miR-4327, and miR-3135b) with noticeable increase in the urine of ESCC patients implied their excellent diagnostic performance [5]. Recent genomics studies have demonstrated the widespread mutational profile of ESCC, involving genes regulating cell cycle, epigenetic process, and certain mutations leading to dysregulation of WNT, NOTCH and receptor-tyrosine kinase PI_3_K signaling pathways [6]. Single nucleotide polymorphism also proved to be connected with ESCC susceptibility, such as rs7447927 at 5q31.2 in *TMEM173* and rs1642764 at 17p13.1 in *ATP1B2* [7]. To a certain extent, these studies contributed to understanding the biological characteristics of ESCC. However, whether these molecules would translate into biomarkers for early diagnosis and screening of ESCC needs further exploration and validation.

Mass spectrometry-based proteomics has emerged as a powerful tool for discovering the potential tumor-associated biomarkers and exploring signaling regulatory networks related to carcinogenesis and progression [8]. Discovering protein biomarkers for diagnosis, unraveling signaling pathway characteristics of ESCC, and developing effective diagnostic and therapeutic strategies will directly benefit the patients. Currently, proteomic based approaches have been applied to identify biomarkers for ESCC. However, most of the proteins mentioned in these studies are single and limited, while their molecular functions have not been fully elucidated [9–11]. Accomplishing in-depth proteomic studies is thus an absolute necessity to explore novel candidate protein biosignature for early diagnosis and further understand the biological characteristics of ESCC.

In this study, we employed quantitative proteomics to characterize the proteome profiles of serum samples in a discovery set containing 30 patients with ESCC and 30 healthy controls. Sixty-nine proteins dysregulated were revealed in ESCC patients and six candidate proteins (S100A8/A9, SAA1, ENO1, TPI1 and PGAM1) were further validated in two independent validation sets. Furthermore, these six candidate proteins in ROC curve analysis exhibited high diagnostic sensitivity and specificity. Pathway enrichment analysis showed that these 69 differentially expressed proteins belong to fifteen major pathways: glycolysis, Cori cycle, folate metabolism, HIF-1*α*, TLR4 signaling, focal adhesion and others. Additionally, our finding displayed that activated glycolysis and TLR4 pathways were positively associated with clinicopathologic TNM stages in ESCC patients.

## Materials and methods

### Subject characteristics

Study subjects included thirty newly-admitted ESCC patients who were untreated and 30 healthy controls. The diagnosis was confirmed by esophagoscopy and biopsy. All the subjects met the following criteria: no individual history of (i) other types of cancer or digestive disease, (ii) active or at high risk of overt bleeding, coagulopathy including haemophilia, (iii) liver dysfunction with impaired coagulation, (iv) treatment with any other investigational agent, and (v) participation in other clinical trials. The clinicopathological staging was estimated based on the 7th edition tumor, lymph node, metastasis (TNM) grading of ESCC. All of the procedures performed in this study involving participants were in accordance with the ethical standards of the institution and the Declaration of Helsinki [12]. All participants signed informed consent prior to participation in the study.

### Sample collection and preparation

The fasting blood from ESCC patients and healthy controls were collected from July 2019 to April 2020 from Affiliated Hospital of North Sichuan Medical College. Serum samples were prepared from the blood by clotting at room temperature for 30 min and centrifuged at 3000 rpm for 10 min. After that, serum aliquots were transferred into sterile Eppendorf tubes and stored at − 80 °C refrigerator until further use. Before using, serum was thawed and pretreated by using the High Select™ TOP 14 Abundant Protein Depletion Mini Spin Columns Kit (catalog number: A36370; Thermo Scientific) to deplete the 14 highest abundance proteins, including albumin, IgG, IgA, IgM, IgD, IgE, kappa and lambda light chains, *α*1-antitrypsin, *α*1-acid glycoprotein, *α*2-macroglobulin, apolipoprotein A1, fibrinogen, haptoglobin, and transferrin, according to manufacturer’s protocol. Simply put, 20 µL 10% sodium deoxycholate and 10 µL of the sample were added to the resin slurry in the depletion spin column. The mixture was incubated for 1 h at room temperature and centrifuged at 1000×*g* for 2 min. The filtrate was collected and transferred into a sterile tube, reduced with 10 mM dithiothreitol at 95 °C for 5 min, and alkylated with 10 mM iodoacetamide at room temperature for 30 min in darkness. Subsequently, 2 µg trypsin was added and incubated at 37 °C for 16 h. Peptides were then loaded on a homemade reverse-phase C18 column in a pipet tip. 293T cell lysate was used to assess the LC-MS/MS stability and reproducibility as a quality control (QC) standard.

### LC-MS/MS analysis

Peptide mixtures were analyzed on an Orbitrap Fusion Lumos (Thermo Fisher Scientific) mass spectrometer interfaced with an Easy-nLC 1200 nanoflow liquid chromatography system (Thermo Fisher Scientific) with a Nono Spray Ionization. Samples were dissolved with 50 µL of Solvent A (0.1% formic acid in water). Following this, 5 µL of the dissolved sample was loaded to a homemade trap column (100 μm × 2 cm) packed with C18 reverse-phase resin (particle size, 3 μm; pore size, 120 Å; SunChrom, USA) at a maximum pressure of 280 bar into which a further 12 µL of solvent A was added. Subsequently, peptides were separated on a 150 μm × 15 cm silica microcolumn (homemade, particle size, 1.9 μm; pore size, 120 Å; SunChrom, USA) with a gradient of 7–32% mobile phase B (100% acetonitrile and 0.1% formic acid) at a flow rate of 600 nL/min for 60 min. The gradient elution conditions were set as follows: 7–10% mobile phase B for 3 min; 10–25% for 39 min; 25–32% for 11 min; 32–95% for 1 min; 95% for 6 min. The MS analysis was performed in a data-dependent manner (DDA) with full scans (*m/z* 350–1500) acquired using an Orbitrap mass analysis at a mass resolution of 120,000, and the automatic gain control (AGC targets) was set to 4e5 with a maximum ion injection time of 50 ms. The most intense ions selected under top-speed mode were isolated in Quadrupole with a 1.6 *m/z* window and fragmented by higher-energy collisional dissociation (HCD) with a normalized collision energy of 32%, then detected in the Orbitrap at a mass resolution of 15,000. The AGC targets for MS/MS were set to 5e4, and the maximum ion injection time was 22 ms. Dynamic exclusion time was set as 30 s.

### Data analysis

Mass spectrometry data were analyzed by MaxQuant (version 1.6.2.10) (http://www.maxquant.org) [13]. MS/MS spectra were searched by the Andromeda search engine against the UniProt-human database (Version 2019.01) supplemented with forward and reverse sequences [14]. Precursor mass and fragment mass were identified in the main Andromeda search engine with an initial mass tolerance of 6 ppm and 20 ppm, respectively. The search included variable modifications of methionine oxidation and *N*-terminal acetylation and fixed modification of carbamidomethyl cysteine. Minimal peptide length was set to seven amino acids, and a maximum of two mis-cleavages was allowed. The false discovery rate (FDR) was set to 0.01 for peptide and protein identifications. For matching, a retention time window of 30 s was selected. In the case of identified peptides shared between two proteins, these were combined and reported as one protein group. Proteins matching the reverse database were filtered out as mismatched proteins. Spearman’s correlation coefficient was calculated for all QC samples.

### Protein quantification and differential protein screening

Proteins were executed based on the following criteria. Potentially contaminated proteins, including keratin, were excluded from the data. Proteins were required to have at least half of the valid values in both groups, and the remaining missing values were imputed with K-nearest neighbors (K = 10) using the R package (v.3.5.2). Protein abundance was quantified using peak area and normalized by each sample’s total area, which represented standardized value of a protein across samples. The standardized value was multiplied by 10^5^ for easy visualization. Fold change (FC) was obtained by calculating the ratio between the standardized average value of each protein in the ESCC group and the healthy group [FC = Average_(ESCC)_/Average_(healthy)_]. Differentially expressed proteins were screened by FC combined with significance levels (*P*-value). Protein abundance was significantly changed if a more than 1.5-fold increase or decrease was observed between two groups with a *P* < 0.05.

### Enrichment analysis

GO enrichment analysis of biological processes was conducted using GSEA software or the Metascape database (https://metascape.org/gp/index.html) [15]. Terms with *P*-value < 0.05, a minimum count of 3 and an enrichment factor of more than 1.5 were collected and grouped into clusters based on their membership similarities (Kappa scores > 0.3) [15]. The most statistically significant term within a cluster was selected to represent the cluster. If more than 20 terms for GO or pathway annotations were identified, the top 20 terms were presented for visualization. Protein–protein interaction (PPI) was executed by Markov Clustering (MCL) with the inflation parameter of more than 3 using the STRING database (https://cn.string-db.org/) [16]. The physical interactions network analysis was accomplished by the Metascape database and networks with scores of more than 0.132 were presented by Cytoscape (v.3.7.2). Pathway enrichment analysis was done using the GeneCard database (https://www.genecards.org/) [17].

### ELISA assays

For validation set, serum S100A8/A9, SAA1, ENO1, TPI1 and PGAM1 of 30 healthy controls and 53 ESCC patients were detected using the Human S100A8/A9 Heterodimer Immunoassay ELISA Kit and Serum Amyloid A1 DuoSet ELISA kit (R&D Systemslnc Minnesota, USA), TPI1 ELISA Kit, ENO1 ELISA Kit and PGAM1 ELISA Kit (Cloud-Clone Corp, China) according to the manufacturer’s instructions. Plates were coated with the capture antibody at room temperature for 1 h and subsequently at 4 °C overnight. After incubation, wells were washed in triplicate by TBST (TBS with 0.05% Tween). Wells were then blocked with 250 µL TBST containing 1% BSA and incubated for 1 h at 37 °C. Diluted samples (100 µL) were incubated with detection antibody for 1.5 h at 37 °C and finally with the HRP for 1 h at 37 ℃ and substrate solution for 30 min at room temperature. The substrate reaction was halted by adding 2 M H_2_SO_4_. Analysis of the optical absorbance was performed by using a plate reader. The absorbance values were determined from the background subtracted from the signal at 450 nm.

### Immunochemistry assays

ESCC tissues and matched adjacent normal tissues (n = 29) were acquired from the Pathology Department of Affiliated Hospital of North Sichuan Medical College. Patients did not receive chemotherapy or radiation before surgery. Immunochemistry was performed as per the standard procedure. Tissue sections were heated at 60 °C for 1 h and then dewaxed in xylene, rehydrated in a graded series of ethanol and incubated in H_2_O_2_ for 5 min at room temperature. Heat-induced epitope retrieval was carried out using Tris-EDTA buffer in a microwave oven heated on high for 3 min and medium for a further 10 min. Sections were cooled at room temperature and then incubated with ENO1, TPI1, PGAM1, SAA1 and S100A8/A9 primary antibody (dilution 1:100) overnight at 4 °C in a humid chamber, followed by incubation with an appropriate secondary antibody. Images were obtained by scanning the slides using a Zeiss Axio Scan Z1 in brightfield mode (Zeiss, Jena, Germany). An IHC score was assigned to each specimen according to the staining intensity and the proportion of positive cells. 1+: light brown and stained cells < 30%; 2+: brown and stained cells 30–60%; 3+: deep brown and stained cells > 60%. Comprehensive scores were made according to the sum of the two indexes. 1+ = weakly positive; 2+ = moderately positive, 3+ = strongly positive. Staining in non-cancer cells was not accounted for in the IHC scores.

### Statistical analysis

Statistical analysis was accomplished using IBM SPSS 23.0 package (IBM SPSS, Turkey). Normality of the data distributions was tested via the Shapiro-Wilks test. The data with normal distribution were analyzed by unpaired Student’s *t*-test. The correlation coefficient was assessed using Pearson and distance correlation analysis. Data were expressed as mean ± SEM, *P* < 0.05 was considered statistically significant. Data were displayed using GraphPad Prism 8.0.1 (GraphPad Prism, Inc. San Diego, USA) or the R package (version 3.5.2). The diagnostic performance of biomarkers was evaluated by calculating ROC curves, AUC, optimal cutoff, sensitivity, specificity, and positive and negative predictive values using the R package. The optimal cutoff value was defined by maximizing Yoden’s index.

## Results

### Cohorts and clinical features of the healthy controls and ESCC patients

A discovery set containing 30 ESCC patients and 30 healthy controls was recruited to characterize the differential proteome. Potential protein biomarkers were further investigated by procuring two independent validation sets, of which validation set_1 included 30 healthy controls and 53 ESCC patients, and validation set_2 included 29 ESCC patients. Serum or tissue samples, including cancerous tissues (CT) and their adjacent normal tissues (ANT), were obtained from each of the subjects. The clinical characteristics of controls and ESCC patients are summarized in Table 1.

**Table 1:** Clinicopathological features of all subjects

Clinic features	Discovery set		Validation set_1		Validation set_2	
	Control (n = 30)	ESCC (n = 30)	Control (n = 30)	ESCC (n = 53)	CT (n = 29)	ANT(n = 29)
Age (mean±SD)	60.1 ± 15.1	64.6 ± 6.9	52.4 ± 10.5	66.8 ± 8.6	63.9±8.8	
Gender						
Male	16	19	19	35	23	
Female	14	11	11	18	6	
T stage						
T1		1		6		
T2		17		22	10	
T3		7		16	18	
T4		5		9	1	
N stage						
N0		8		21	13	
N1		12		18	8	
N2		6		8	6	
N3		4		6	2	
M stage						
M0		26		48	25	
M1		4		5	4	
Overall stage						
I		2		8		
II		16		28	16	
III		6		12	10	
IV		6		5	3	

### Quantification of proteomic profiling of ESCC and healthy controls

**Fig. 1:**
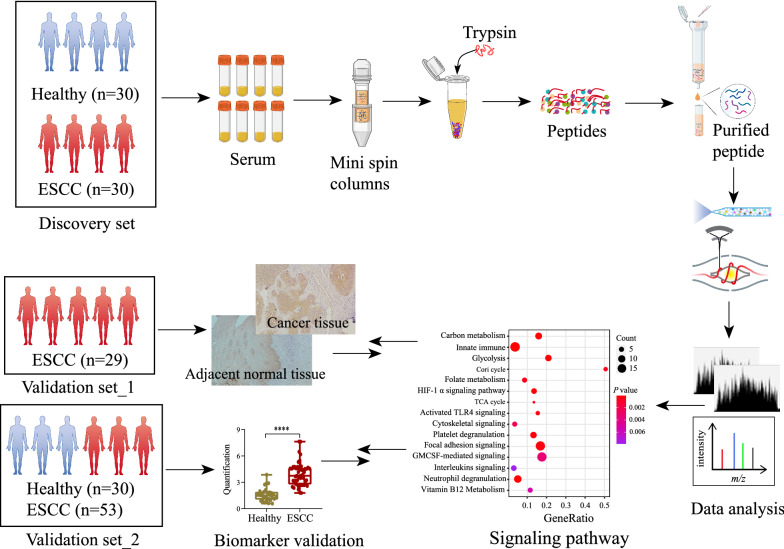
A workflow of the experiment based on serum proteomics

Serum proteomics-based integrated function validation approach was employed to identify potential ESCC-associated protein biomarkers and uncover functional characteristics in our study. A brief description of the workflow is shown in Fig. 1. Proteins extracted from ESCC and healthy controls were processed using High Select Protein Depletion Mini Spin Columns to remove the 14 highest abundant proteins. Then proteins were digested by trypsin, after which peptides were separated by the Reverse-phase C18 column, and subsequently followed by LC-MS/MS analysis. Differential proteins screening and functional enrichment analysis were accomplished by bioinformatics analysis. Then we employed two independent validation sets to ascertain identified ESCC-associated biomarkers by ELSA and IHC.

**Fig. 2:**
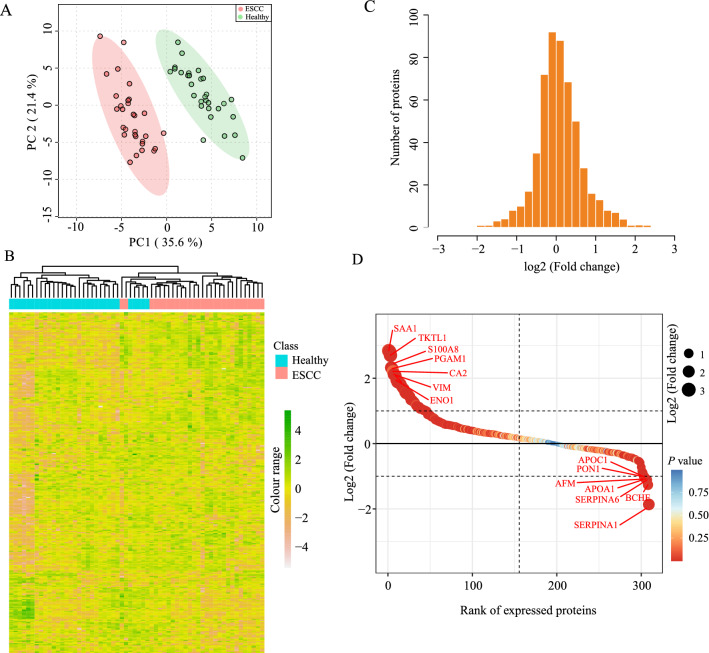
Proteomic profiling of ESCC patients and healthy controls. **A** Principal component analysis in both groups. **B** Heatmap analysis of expression profile of proteins. **C** The distribution of protein abundance ratios. The fold changes of ESCC/control are shown in log_2_ scale on the x-axis, and the numbers of proteins are shown on the y-axis. **D** Rank plots show the top increased and decreased proteins in the ESCC group

The mass spectrometry proteomics data have been deposited to the ProteomeXchange Consortium (http://proteomecentral.proteomexchange.org) via the iProX partner repository with the data identifier PXD031287. A total of 633 non-redundant proteins were identified with at least two unique peptides at 1% peptide level FDR. In this pool, 310 proteins detectable in at least half of all samples were selected for subsequent bioinformatics analysis (Additional file 2: Table S1). The high positive correlation (*r ≥* 0.90) of the peak area in each QC sample displayed good repeatability (Additional file 1: Fig. S1). Healthy controls and ESCC patients were completely separated, as shown by principal component analysis (PCA), and each group of samples showed well-clustering based on the first two principal components (Fig. 2A). Next, the heatmap visualized the whole proteome comparison between the two groups, which indicated a significant change (Fig. 2B). The distribution of protein abundance ratio displayed by histogram obeyed normal distribution (Fig. 2C). The top increased proteins exhibited by the protein rank plot were SAA1, TKTL1, S100A8, PGAM1, CA2, VIM and ENO1, while the proteins decreased were SERPINA1/6, BCHE, APOA1, AFM, PON1 and APOC1 (Fig. 2D).

### Identification of differentially expressed proteins

**Fig. 3:**
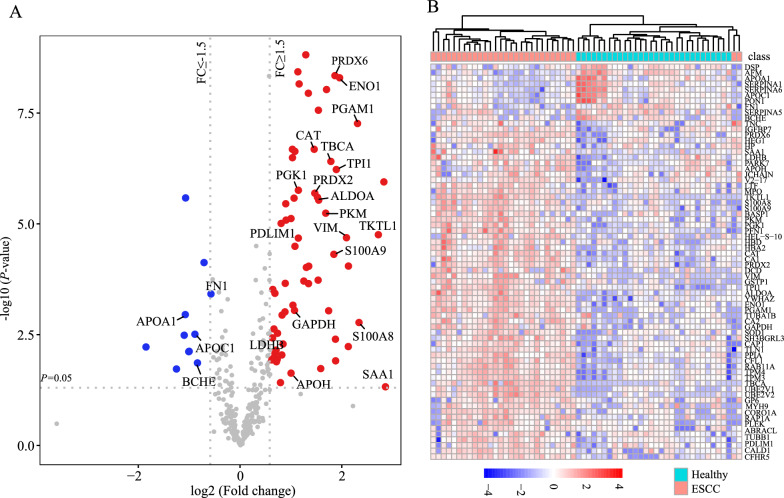
Bioinformatics analysis of differentially expressed proteins. **A** Differential proteins are presented by the volcano plot. The vertical dotted lines represent proteins with a more than 1.5-fold increase (marked with red) or decrease (marked with blue), respectively. The gray dots are considered as no significant change, and horizontal dotted lines display cutoff *P-*value. **B** Heat map visualization of 69 differential proteins. The increased and decreased proteins are represented by a range of red and blue intensities, respectively

A volcano plot was applied to delineate differential proteins abundance against the corresponding *P-*value obtained from the *t-*test (Fig. 3A). The quantitative values of 59 proteins (19.0% of the proteome) and 10 proteins (3.26% of the proteome) displayed a more than 1.5-fold increase (marked as red dots) or decrease (marked as blue dots) in ESCC samples compared with controls (Additional file 2: Table S2). The rest of the 241 proteins (77.74% of the proteome) abundance was regarded as no significant change (marked as gray dots). Furthermore, the heatmap of hierarchical cluster analysis indicated that most of the 69 differentially expressed proteins increased distinctively in the ESCC group (Fig. 3B).

### Enrichment analysis and signaling pathway analysis

**Fig. 4:**
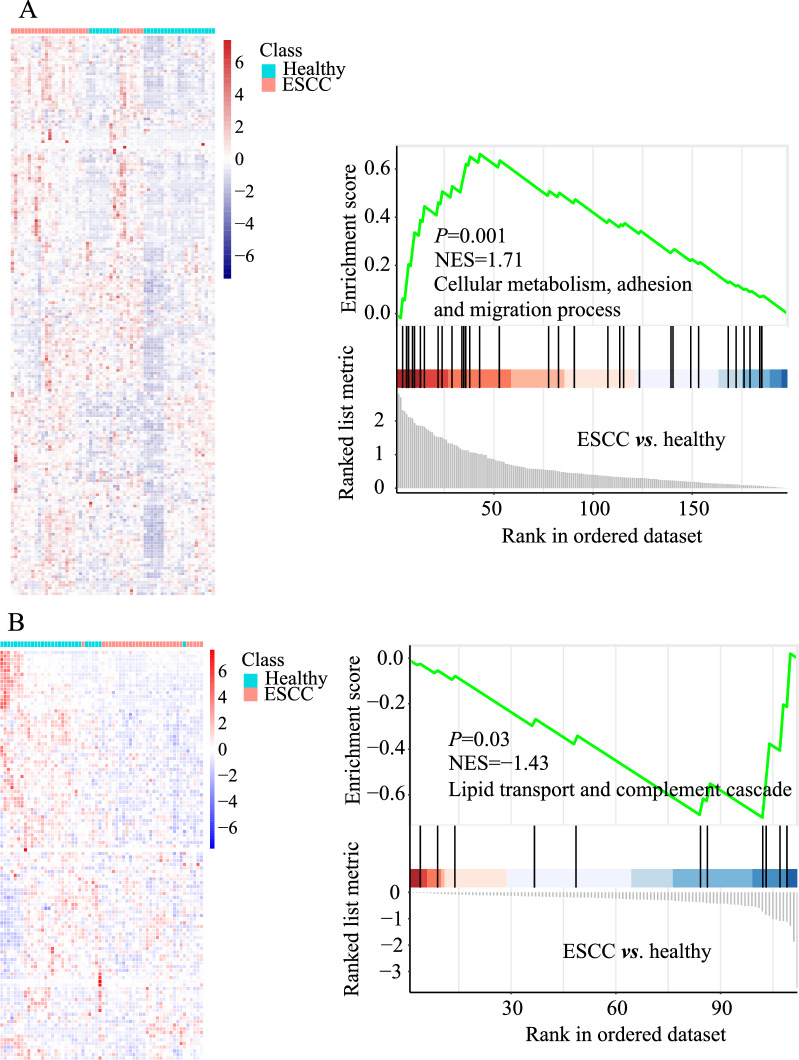
Heatmap and GSEA analysis of increased (**A**) and decreased (**B**) proteins

**Fig. 5:**
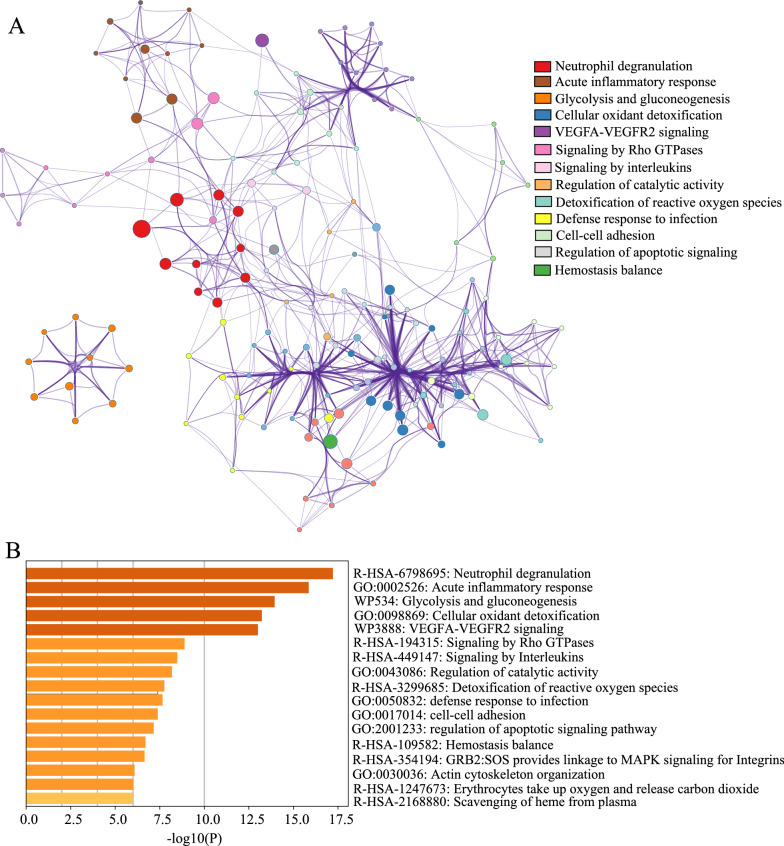
Gene ontology enrichment analysis of increased and decreased protein. Enrichment results are displayed by network plot (**A**) and *P-*value (**B**), respectively. Sizes of nodes correspond to the degree of enrichment

**Fig. 6:**
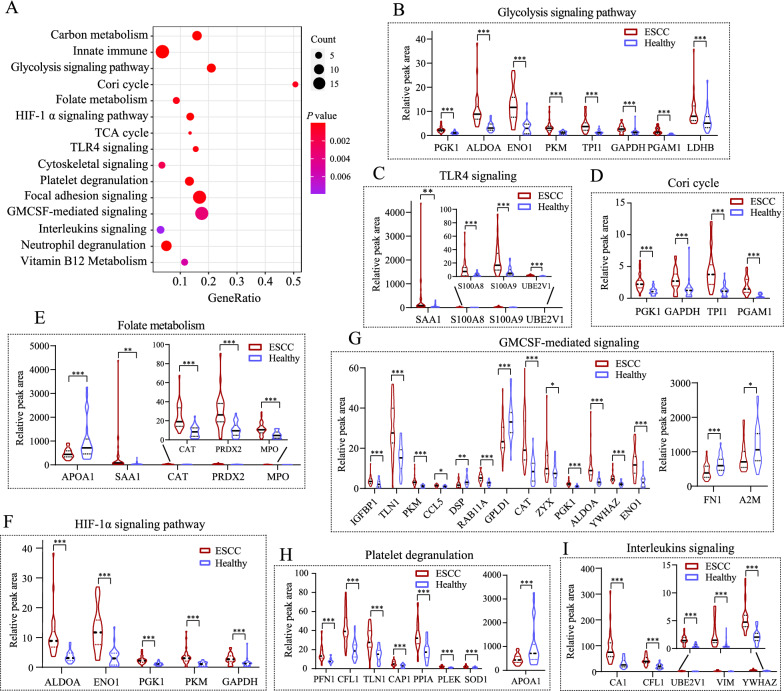
System-wide analysis of signaling pathways of ESCC patients. **A** Signaling pathway analysis of differential proteins by using the GeneCard dataset. Size of the nodes shows the number of matched proteins, color of the nodes represents *P-*value of the enrichment analysis. **B**–**I** Quantitative analysis of glycolysis, TLR4, Cori cycle, folate metabolism, GMCSM, HIF-1α, platelet degranulation and interleukins signaling pathway in two groups, respectively. Two-tailed *t*-test is performed, *P < 0.05, **P < 0.01, and ***P < 0.001 vs. controls

To gain a comprehensive understanding of the biological significance of these 310 proteins, we conducted GSEA to characterize their potential functions. Our data showed that all increased proteins (FC > 1.0) in the ESCC group were moderated associated with cellular metabolism, adhesion and migration process (NES = 1.71, *P* = 0.001), whereas decreased proteins (FC < 1.0) were involved in lipid transport and complement cascade (NES = −1.43, *P* = 0.03) (Fig. 4A, B), respectively. Furthermore, up-and down-regulated proteins (FC > 1.5 or < 0.67 and *P* < 0.05) were subjected to GO terms enrichment analysis by the Metascape database, and the results were presented in Fig. 5A, B, respectively. Those proteins were discerned to be related to a series of biological events (Fig. 5A) and remarkably enriched in neutrophil degranulation (−log_10_*P* = 17.22), acute inflammatory response (−log_10_*P* = 15.86) and glycolysis (−log_10_*P* = 13.94), and many others. (Fig. 5B). Signaling pathway analysis of differentially expressed proteins was performed by the GeneCard database. GeneRatios were calculated via analyzing matched proteins and the corresponding *P* values according to FDR. Consequently, the top 15 signaling pathways were visualized by a bubble chart (Fig. 6A). Cori cycle showed the highest enrichment ratio with a value of 0.5 among all pathways, whereas glycolysis displayed the lowest *P*-value (*P* = 1.06E−09) (Table 2). Correspondingly, pathways of carbon metabolism, innate immune, HIF-1*α* signaling, TLR4 activation, and focal adhesion showed remarkable changes, indicating the dysfunction of multiple signaling pathways in ESCC patients. Proteins involved in glycolysis, TLR4 signaling, Cori cycle, folate metabolism, GMCSF-mediated signaling, HIF-1*α* pathway, platelet degranulation, and interleukins signaling, were increased accordingly in ESCC groups (Fig. 6B–I).

**Table 2:** Significantly altered signaling pathways based on differentially expressed proteins in ESCC group

Description	*P* value	GeneRatio	Count
Carbon metabolism	1.59E−09	0.16	9
Innate immune	1.03E−08	0.03	19
Glycolysis	1.06E−09	0.21	8
Cori cycle	5.12E−04	0.50	4
Folate metabolism	3.42E−04	0.08	5
HIF-1*α* signaling pathway	1.10E−05	0.13	6
TCA cycle	3.30E−04	0.13	3
Activated TLR4 signaling	1.80E−04	0.15	4
Cytoskeletal signaling	2.16E−03	0.03	5
Platelet degranulation	1.20E−04	0.13	8
Focal adhesion signaling	2.30E−04	0.16	18
GMCSF-mediated signaling	3.40E−03	0.17	18
Interleukins signaling	7.90E−03	0.03	6
Neutrophil degranulation	1.13E−06	0.05	11
Vitamin B12 metabolism	5.20E−03	0.11	5

### Protein–protein interaction enrichment analysis

**Fig. 7:**
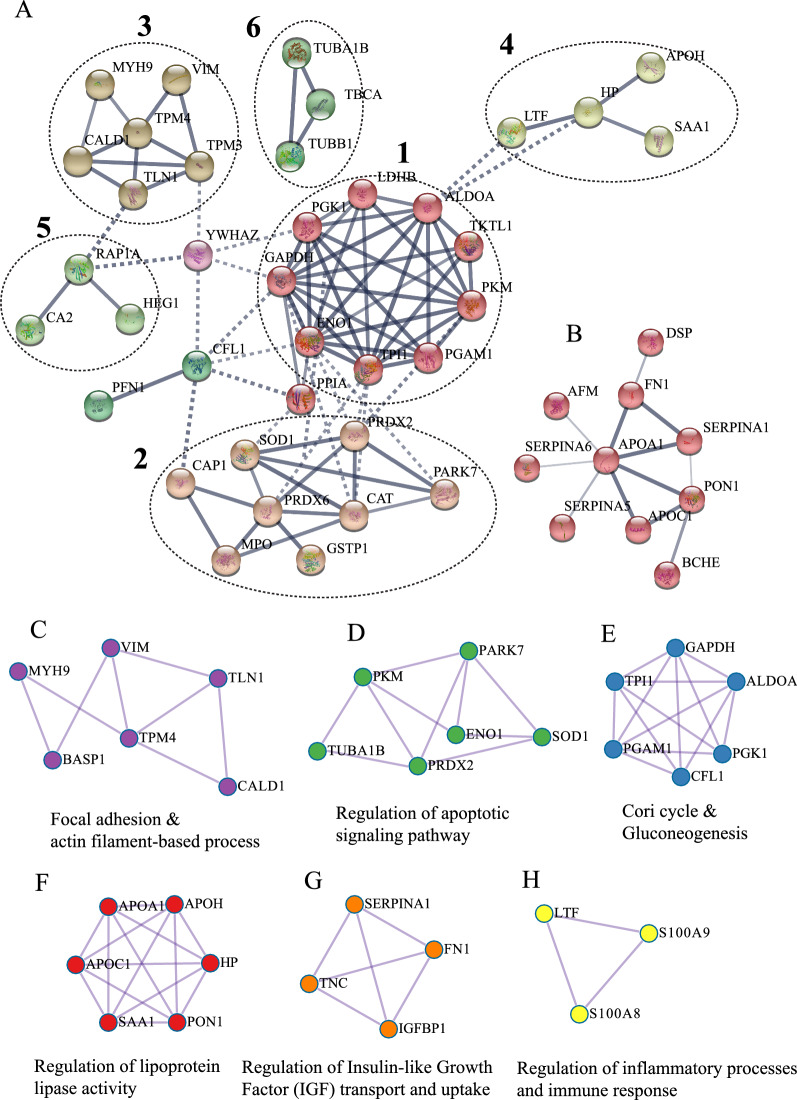
Network analysis of differentially expressed proteins. **A**, **B** Protein–protein interaction networks of up-and down-regulated proteins by Markov Clustering based on STRING database, respectively. Each circle represents a sub-network. **C**–**H** Physical interactions analysis of differential proteins by Molecular Complex Detection by using Metascape database (scores > 0.132)

The STRING database was employed to construct the protein–protein interaction networks among these 69 proteins. First, we used Markov Clustering to cluster the network with an inflation parameter of more than 3. The results suggested that the up-regulated protein network consisted of six sub-networks (Fig. 7A), of which sub-network 1 occupied the dominant position of the entire network consisting of ENO1, GAPDH, PGAM1, PKM, ALDOA, LDHB, PGK1, TPI1 and TKTL1, the biological functions of these molecules were related to glucose metabolism. Sub-network 2 consisted of eight regulated proteins (SOD1, PRDX2, CAP1, PRDX6, CAT, PARK7, MPO and GSTP1), which played an important role in antioxidant activity. Sub-network 3 was comprised of six molecules (VIM, MYH9, TPM4, TPM3, CALD1 and TLN1), which were involved in focal adhesion. Sub-networks 4 was made up of four proteins (APOH, HP, SAA1 and LTF), which were associated with acute inflammatory response, and sub-network 5 and 6 were formed by three regulated proteins respectively, which primarily were associated with response to carbohydrate (RAP1A, CA2 and HEG1 for sub-network 5) and tubulin folding pathway (TUBA1B, TBCA and TUBB1 for sub-network 6). In addition, we noticed that APOA1, APOC1, SERPINA1/5/6, PON1, BCHE, FN1, DSP and AFM formed primary hub molecules of downregulated proteins (Fig. 7B). The biological functions of these molecules were related to glucocorticoid biosynthesis, metabolic process, cholesterol homeostasis and lipid metabolism. Next, we constructed physical interactions (scores > 0.132) to identify densely connected network molecules by Molecular Complex Detection (MCODE) using the Metascape database with the final accomplished MCODE networks shown in Fig. 7C–H. Consistent with the above results, our data showed that six sub-networks were formed from these up-and down-regulated proteins, including focal adhesion and actin filament-based process, Cori cycle and gluconeogenesis, regulation of apoptotic signaling pathway, lipoprotein lipase activity, insulin-like growth factor transport and uptake, and inflammatory processes and immune response.

### Validation of ESCC-related candidate biomarker panel

**Fig. 8:**
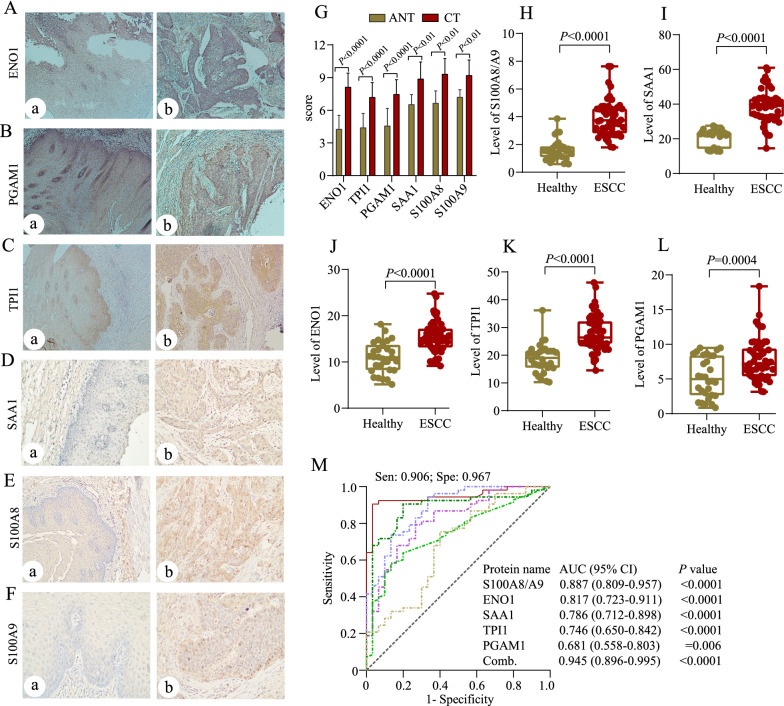
Validation of ESCC-related candidate biomarker panel. **A**–**F** Representative images of carcinoma tissue and their ANT stained for ENO1, TPI1, PGAM1, SAA1 and S100A8/A9 (brown) using immunohistochemistry assay (×100), respectively. **a** NAT and **b** CT. **G** Scores from staining intensity and numbers in 29 pairs of brown cells in cancer and adjacent normal tissues. **H**–**L** Quantification analysis of S100A8/A9, SAA1, ENO1, TPI1 and PGAM1 in serum from healthy controls and ESCC patients. **M** ROC curve for serum against SAA1, S100A8/A9, ENO1, TPI1, PGAM1 and their combination for patients with ESCC versus normal controls. *ANT* adjacent normal tissue, *CT* carcinoma tissue

The candidate biomarkers were validated based on proteomics data and fold changes from ESCC patients and healthy controls. Subsequently, we quantified ENO1, TPI1, PGAM1, SAA1and S100A8/A9 of cancerous tissues and their matched adjacent normal tissues in an independent validation set including 29 ESCC patients by immunohistochemistry. The relevant clinical information is summarized in Table 1. In line with proteomics results, significantly increased expression of these six proteins were observed in cancerous tissues (Fig. 8A–G). In another validation set, a batch of serum samples of 30 healthy controls and 53 ESCC patients were collected to verify their levels by ELISA. The results showed that the mean concentrations of S100A8/A9 were (3.92 ± 1.27) and (1.59 ± 0.73) µg/mL, SAA1 (39.06 ± 9.23) and (20.68 ± 4.79) µg/mL, ENO1 (15.46 ± 3.48) and (11.02 ± 3.25) µg/mL, TPI1 (27.98 ± 6.46) and (18.85 ± 5.23) µg/mL, as well as PGAM1 (7.78 ± 3.01) and (5.27 ± 2.84) µg/mL in ESCC and healthy groups, respectively (Fig. 8H–L). It came as no surprise that the ROC curve analysis also confirmed the fairly high sensitivity and specificity of these combined six proteins for distinguishing ESCC patients from controls (sensitivity: 0.906 and specificity: 0.967, respectively, with an AUC of 0.945), which is clearly greater than that of S100A8/A9 (AUC: 0.887), SAA1 (AUC: 0.786), ENO1 (AUC: 0.817), TPI1 (AUC: 0.746) and PGAM1 (AUC: 0.681) alone.

### Activated glycolysis and TLR4 signaling pathways are relevant to clinicopathological TNM stages in ESCC patients

**Fig. 9:**
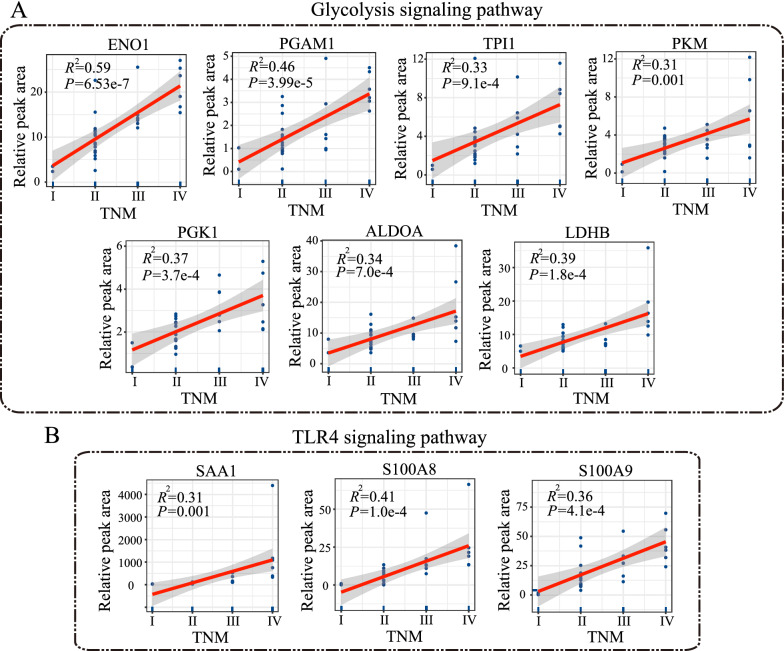
Pearson correlation analysis between proteins-related glycolysis (**A**), TLR4 signaling pathway and TNM stages, respectively

The protein expression of glycolysis increased in ESCC patients (Fig. 6B), and among them, shown by Pearson correlation analysis, levels of ENO1, PGAM1, TPI1, PKM, PGK1, ALDOA and LDHB were positively correlated with clinicopathological TNM stages (*R*^2^ = 0.59, *P* = 6.53e−7, *R*^2^ = 0.46, *P* = 3.99e−5, *R*^2^ = 0.33, *P* = 9.1e−4, *R*^2^ = 0.31, *P* = 0.001, *R*^2^ = 0.37, *P* = 3.7e−4, *R*^2^ = 0.34, *P* = 7.0e−4, and *R*^2^ = 0.39, *P* = 1.8e−4, respectively) (Fig. 9A). We observed notable increase of proteins in TLR4 signaling pathway, including SAA1, S100A8 and S100A9 (Fig. 9B), which were positively correlated with clinicopathological TNM stages (*R*^2^ = 0.31, *P* = 0.001, *R*^2^ = 0.41, *P* = 1.0e-4, and *R*^2^ = 0.36, *P* = 4.1e-4, respectively).

## Discussion

Blood biomarkers are widely applied for the screening and diagnosis of tumor because of their ready availability. Blood contains a range of proteins with concentrations up to 12 to 13 orders of magnitude, while 90% of low concentration proteins are masked by a few high-abundant ones, such as albumin and immunoglobulins. Due to this, although these low-abundance proteins may contain critical information regarding the disease status, they are hard to be detected and quantified in proteomics. In this study, we employed the High Select TOP 14 Abundant Protein Depletion Mini Spin Columns to deplete the 14 highest abundant proteins, which contributes to the in-depth detection of ESCC-related proteins, including low abundance proteins, as well as provides data support for biomarker discovery and signaling pathway research.

Traditional biomarkers, such as carcinoembryonic antigen (CEA), carbohydrate antigen 19-9 (CA 19-9), as well as squamous cell carcinoma antigen (SCCA), showed low sensitivity and specificity to the diagnosis of ESCC [18]. Besides, the signaling regulatory networks associated with ESCC pathogenesis and poor prognosis have not been fully elucidated. Therefore, finding valid biomarkers to identify early stages of ESCC progression and unraveling signaling pathways mediated by these molecules hold great promise for providing potential novel targets for ESCC therapy.

S100A8 and S100A9, known as calcium-binding proteins, belong to the S100 family. They usually exist in the form of heterodimers for stability. S100A8/A9, being multifunctional, can induce recruitment of leukocytes, promotion of cytokine and chemokine production, and regulation of leukocyte adhesion and migration [19]. Its intracellular functions include facilitating leukocyte arachidonic acid trafficking and metabolism, modulation of the tubulin-dependent cytoskeleton, and activation of the neutrophilic NADPH-oxidase [20, 21], and extracellular functions involve pro-inflammatory [20], oxidant-scavenging and apoptosis-inducing activities [22]. Several studies have concluded that S100A8/A9 could be released by tumor cell necrosis following hypoxia within growing tumors [23]. Regardless of the source, S100A8/A9 appears to play essential roles in both inflammation-induced cancer and cancer-induced inflammation and mediate concentration-dependent protumor response [23]. Previous research demonstrated that S100A8/A9 is involved in the occurrence and progression of tumors, and its high level is strongly associated with poor prognosis [24]. It can also be considered a potential tumor biomarker for melanoma and hepatocellular carcinoma [24, 25].

Nevertheless, other studies suggested that S100A8 and S100A9 were significantly downregulated in the tissues of ESCC patients compared with controls at the mRNA level [26, 27]. Since transcription level is shown to have a poor coherence with protein expression, without doubt, measurement of protein is more efficient than detecting mRNA for mining actionable biomarkers. Contradictory with the previous reports, our results indicated that S100A8/A9 was significantly up-regulated in both serum and tissue of ESCC patients compared with controls and exhibited high sensitivity and specificity for distinguishing ESCC patients from controls with an AUC of 0.887. From the perspective of regulating signaling pathways, S100A8/A9 acts as an alarming or a danger associated pattern molecule and stimulates innate cells via binding to pattern recognition receptors such as TLR4 [28], then it activates MAPK and NF-*κ*B signaling pathways resulting in the amplification of the pro-inflammatory cascade [29]. In accordance with it, significant alteration of multiple signaling pathways regulated by S100A8/A9 was unearthed in our study, including TLR4, innate immune, as well as neutrophil degranulation. In addition, our findings indicated that S100A8/A9 levels were strongly positively correlated with TNM stages with *R*^2^ = 0.41, *P* = 1.0e−4 and *R*^2^ = 0.36, *P* = 4.1e−4, respectively.

SAA1 is recognized as a nonspecific, acute-phase protein secreted in response to inflammation-associated cytokines, such as interleukin-1/6 (IL-1/6) and tumor necrosis factor *α* (TNF*α*). It has been investigated in various malignancies as a predictor of cancer risk and prognostic factor [30, 31]. A previous study demonstrated that serum SAA1 levels increased in ESCC patients compared with healthy subjects, which may be considered an independent and vital prognostic indicator for patients with ESCC following curative esophageal resection [32]. SAA1 showed a good diagnostic performance in our data (sensitivity: 0.70 and specificity: 0.857, respectively) for distinguishing patients with ESCC from controls. SAA1 levels were also found to be positively correlated with unfavorable TNM stages closely linked with cancer progression and poor prognosis.

SAA1 is produced by the liver and enters the systemic circulation in response to stimulation by inflammatory cytokines, such as IL-6. As a potent pro-inflammatory cytokine, IL-6, stimulates the liver to produce SAA1 [29]. Elevated IL-6 in serum has been shown to be related to disease progression and poor prognosis in esophageal cancer [33]. Although our data did not detect IL-6 directly, pathway enrichment analysis revealed that the interleukin signaling pathway was activated, and levels of related molecules were significantly increased in ESCC serum, including CA1, CFL1, UBE2V1, VIM and YWHAZ, which mediate the expression of IL-3, IL-4, IL-5, IL-12 and IL-13. Based on these results, we speculated that the increased SAA1 might be due to excessive inflammatory cytokines, which have previously been proven to be produced by cancer cells in patients with advanced esophageal carcinoma [33, 34].

Our proteomics data showed 5.01, 3.55, 7.18, 3.84, 3.67 and 4.90-fold higher levels of S100A8, S100A9, SAA1, ENO1, TPI1 and PGAM1 in ESCC patients sera compared with controls, respectively. Given the close relationship between S100A8/A9, SAA1, ENO1, TPI1, PGAM1 and tumor, with our present data, we were committed to developing a biomarker panel consisting of these six proteins for early detection of ESCC patients, which have yielded high specificity and sensitivity of 0.906 and 0.967, and AUC up to 0.945, higher than each of them alone, suggesting the synergistic effects and increased diagnostic efficacy of these six biomarkers as a potential biomarker panel.

ENO1, a key glycolytic enzyme, which may play a pivotal role in aerobic glycolysis, contributed to solid tumor progress. Evidence showed that ENO1, whose overexpression was associated with multiple tumors, is explained as a key protein in tumorigenesis, proliferation, metastasis and poor outcomes [35, 36]. However, ENO1 expression is more diversified in esophageal cancer than in others. Its expression was abnormally elevated in ESCC and EAC cancerous tissues compared with adjacent non-cancerous tissue. Unexpectedly, significantly lower ENO1 in plasma was found in EAC patients compared to normal subjects, and neither local nor systemic ENO1 levels were significantly associated with overall survival. There was no significant difference in ENO1 detected between ESCC and EAC patients in situ protein levels, suggesting no association of ENO1 expression with the pathological tumor type [37]. Our findings, which are different from other studies, displayed that ENO1 concentrations were significantly increased in both serum and cancerous tissues of ESCC patients, which were strongly positively correlated with the clinicopathological TNM stages (*R*^2^ = 0.59, *P* = 6.53e−7). Overall, these results suggested that ENO1 played different biological roles depending on the types of cancer while the exact mechanisms of action remain unclear.

TPI1 is a metabolic enzyme that catalyzes the interconversion between dihydroxyacetone phosphate and glyceraldehyde-3-phosphate in glycolysis and gluconeogenesis. Elevated TPI1 expression is associated with high levels of metabolism required for rapid tumor growth. A previous study showed a higher expression of TPI1 in metastatic ovarian tumors than in primary ovarian cancers. However, the expression of TPI1 was lower in metastatic cervical tumors than in primary cervical cancers [38]. It was also shown to be significantly increased in intrahepatic cholangiocarcinoma tissues and correlated with a higher recurrence rate and has the potential to act as a novel candidate biomarker for predicting the recurrence of intrahepatic cholangiocarcinoma [39]. These results suggested that TPI1 may play pluralistic biological roles in the development and metastasis of different cancers. However, TPI1 has not been comprehensively reported in ESCC so far, including its clinical significance, biological functions, and underlying molecular mechanisms. Notably, our data showed a significant elevation of TPI1 in ESCC patients with its level being positively correlated with tumor TNM stage (*R*^2^ = 0.33, *P* = 9.1e−4).

PGAM1 is known as a metabolic enzyme in glycolysis. High expression of PGAM1 is closely correlated with lymphatic metastasis and tumor re-occurrence, which might account for the poor prognosis [40]. We found an upregulation of PGAM1 up to 5-fold in the serum with an overexpression in tissues of patients with ESCC. These results indicated a positive correlation of PGAM1 with unfavorable clinicopathological TNM stages (*R*^2^ = 0.46, *P* = 3.99e−5), thereby potentiating the role of PGAM1 as a diagnostic and prognostic marker for ESCC. Furthermore, several proteins related to glycolysis, such as PKM, PGK1, ALDOA and LDHB, were significantly elevated in the serum of ESCC patients and positively correlated with TNM stages (*R*^2^ = 0.31, *P* = 0.001, *R*^2^ = 0.37, *P* = 3.7e−4, *R*^2^ = 0.34, *P* = 7.0e−4, and *R*^2^ = 0.39, *P* = 1.8e−4, respectively), which suggested activated glycolysis could be a significant characteristic of ESCC.

It is widely known that PGK1, ALDOA, ENO1, PKM, TPI1, GAPDH, PGAM1 and LDHB are key regulators in glycolysis and are mainly located in the cytoplasm. However, recent studies detected several glycolysis-related proteins in the extracellular region, such as serum [41–43], of which serum-derived exosomes are the primary source of these proteins. To our knowledge, glycolytic molecules expression levels and their relationship with TNM stages have not been widely reported in serum proteomics of ESCC patients. Undoubtedly, our findings highlighted the importance of glycolysis in ESCC and provided novel insights into pathogenesis for ESCC. In addition, previously published data proposed that hypoxia-inducible factor (HIF) enhanced glycolysis by increasing the transcription of glycolytic enzyme genes to protect cancer cells from energy starvation [44]. At the same time, the activated HIF-1α signaling pathway was demonstrated in current data, which was consistent with previous reports.

## Conclusions

Our study comprehensively portrayed serum proteomic profiling of ESCC patients, identified 69 differential proteins and systematically elucidated multiple abnormal signaling pathways in ESCC patients. Our data showed a potential biomarker panel composed of six protein molecules, including S100A8/A9, SAA1, ENO1, TPI1 and PGAM1, with high diagnostic sensitivity and specificity and revealed the relevance of activated glycolysis and TLR4 pathways to TNM stages. These results propose potential biomarkers for early screening and diagnosis of ESCC and shed new light on the biological characteristics of ESCC from the perspective of signaling pathway.

## Supplementary Information

**Additional file 1: Figure S1.** Spearman’s correlation coefficients from QCs samples to assess the experiment reproducibility of control and ESCCgroups.

**Additional file 2: **Quantitative proteins in ESCC and Control groups.

## Data Availability

The mass spectrometry data is available through the ProteomeXchange consortium with the dataset identifier PXD031287.

## Abbreviations

ESCC: Esophageal squamous cell carcinoma
CT: Cancerous tissues
ANT: Adjacent normal tissues
GO: Gene ontology
GSEA: Gene Set Enrichment Analysis
ELISA: Enzyme-linked immunosorbent assay
IHC: Immunohistochemistry
ROC: Receiver operating characteristics
AUC: Area under characteristic curve
PFN2: Profilin 2
HOTAIR: HOX transcript antisense RNA
TNM: Tumor, lymph node, metastasis
QC: Quality control
DDA: Data-dependent manner
HCD: Energy collisional dissociation
FDR: False discovery rate
PPI: Protein–protein interaction
PCA: Principal component analysis
MCODE: Molecular Complex Detection
CEA: Carcinoembryonic antigen
SCCA: Squamous cell carcinoma antigen
HIF: Hypoxia inducible factor
FC: Fold change
