# Supplementary material for: Serum proteomics unveil characteristic protein diagnostic biomarkers and signaling pathways in patients with esophageal squamous cell carcinoma
Source: Clin Proteomics. 2022 May 24;19:18. doi: 10.1186/s12014-022-09357-x (PMC9128263; doi:10.1186/s12014-022-09357-x)
Supplement: Supplementary file 1 — Additional file 1: Figure S1. Spearman’s correlation coefficients from QCs samples to assess the experiment reproducibility of control and ESCCgroups. [file 12014_2022_9357_MOESM1_ESM.docx]

Serum proteomics unveil characteristic protein diagnostic biomarkers and signaling pathways in patients with esophageal squamous cell carcinoma

Wenhu Liu^#1^, Qiang Wang^#2^, Jinxia Chang^1^, Anup Bhetuwal^3^, Nisha Bhattarai^4^, Fan Zhang^*1^, Jiancai Tang^*1^

^1^ School of Pharmacy, School of Basic Medical Sciences & Forensic Medical, North Sichuan Medical College, Nanchong, China

^2^ Department of Clinical Laboratory, Translational Medicine Research Center, Affiliated Hospital of North Sichuan Medical College, Nanchong, China

^3^ Sichuan Key Laboratory of Medical Imaging, North Sichuan Medical College, Nanchong, China

^4^ Department of Neurology, North Sichuan Medical College, Nanchong, China

^*^Corresponding author: F. Zhang, [zhangfan596@163.com](mailto:zhangfan596@163.com); J. Tang, [tangjiancai@nsmc.edu.cn](mailto:tangjiancai@nsmc.edu.cn)

^#^ These authors contributed equally.

Additional figure


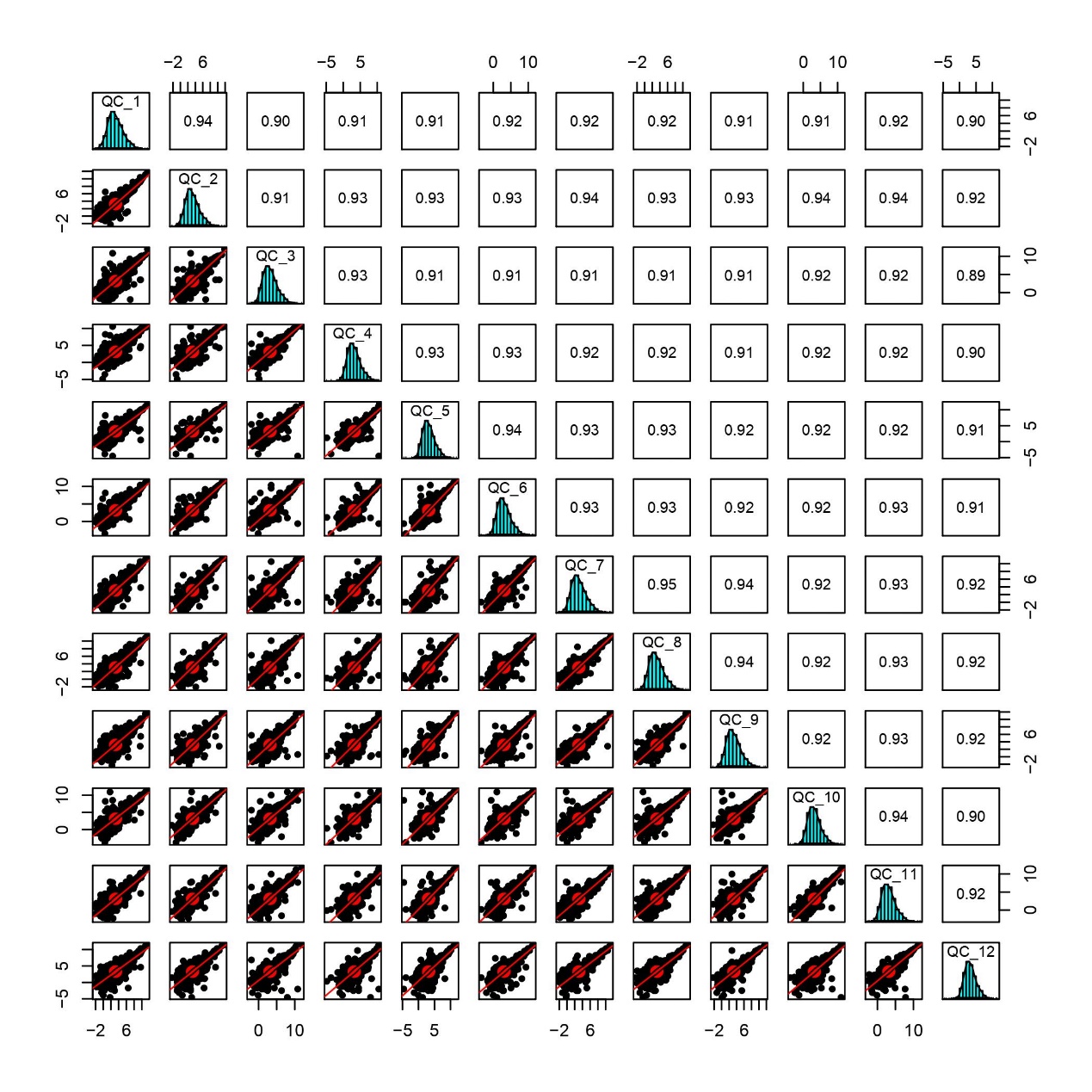


Figure S1: Spearman’s correlation coefficients from QCs samples to assess the experiment reproducibility of control and ESCC groups. The lower-left half shows pairwise scatter plots of biological repeats. The upper-right half shows pairwise Spearman’s correlation coefficients for the same comparison.
